# Glomerular filtration rate change during chronic hepatitis C treatment with Sofosbuvir/Ledipasvir in HCV/HIV Coinfected patients treated with Tenofovir and a boosted protease inhibitor: an observational prospective study

**DOI:** 10.1186/s12879-018-3278-3

**Published:** 2018-08-03

**Authors:** Cristina Aurora São Pedro Soeiro, Celina Andreia Melo Gonçalves, Marta Sofia Correia Marques, Maria Josefina Vazquez Méndez, Ana Paula Ribeiro Almeida Tavares, Ana Maria Lacerda Morgado Fernandes de Carvalho de Aboim Horta, Rui Manuel do Rosário Sarmento-Castro

**Affiliations:** Infectious Diseases Department, Centro Hospitalar do Porto, Largo do Prof. Abel Salazar, 4099-001 Porto, Portugal

**Keywords:** Co-infection HIV/HCV, HCV treatment, Sofosbuvir/ledipasvir, Renal toxicity, Tenofovir, Protease inhibitor, Drug-drug interactions

## Abstract

**Introduction:**

Concomitant use of ledipasvir and boosted protease inhibitors (PIs) may increase the risk of tenofovir (TDF) nephrotoxicity, since both these drugs increase TDF levels. Our aim was to evaluate glomerular filtration rate (eGFR) evolution during HCV treatment with sofosbuvir/ledipasvir (SOF/LDV) in HCV/HIV coinfected patients, according to their antiretroviral treatment (ARV).

**Methods:**

Observational prospective study of HCV/HIV coinfected patients treated with SOF/LDV. eGFR evolution was evaluated during and 12 weeks after HCV treatment. Patients were categorized in three groups based on ARV regimen: non TDF, non-boosted TDF and TDF + boosted PI.

**Results:**

We included 273 patients: 145 were receiving a non-TDF regimen, 78 a non-boosted TDF scheme and 50 were receiving TDF + boosted PI. We observed a statistically significant decrease in eGFR during treatment in all groups (non TDF *p* = 0.03, 95%CI [0.23–3.86], non-boosted TDF *p* < 0.01, 95%CI [3.36–7.44], TDF + PI *p* = 0.01, 95%CI [1.09–7.53]). The decrease was more pronounced in those receiving unboosted TDF (− 5.40 ml/min/1.73m^2^), but differences in eGFR decrease between the three groups were small and not statistically different (*p* = 0.06). eGFR decrease was greater in patients treated for 24 weeks (*p* = 0.009) and in cirrhotic patients (*p* = 0.036). At the end of follow up a recovery of eGFR was observed in all groups.

**Conclusion:**

We observed a significant decrease in eGFR during treatment in all study groups, that was small and reversible after SOF/LDV discontinuation. TDF was not associated with an increase in renal toxicity.

## Background

According to the World Health Organization (WHO), 2–15% of the people infected by the human immunodeficiency virus (HIV) are coinfected with hepatitis C virus (HCV), and this percentage goes up to 90% in those who acquired the infection through intravenous drug use [1]. Liver disease is currently one of the leading causes of morbidity and mortality in HIV infected people [2, 3] and HIV coinfected patients seem to have a faster progression to cirrhosis, a higher rate of liver decompensation and liver death and a lower response to HCV therapy [4–6] . However, HCV treatment has been shown to decrease the risk of negative outcomes even in co-infected patients [7].

With the advent of direct acting antivirals (DAAs), the sustained virologic response (SVR) rates in mono and HIV co-infected patients are similar. With these agents, high cure rates are achieved in the co-infected population, with excellent tolerability and convenient posology [8, 9]. HIV specialists have always been aware of the importance of surveilling and managing drug-drug interactions (DDIs) between antiretroviral medication (ARV) and other co-medication. DDIs are currently one of the problems in the use of DAAs in co-infected patients [8–10]. Clinicians are advised to check possible DDIs between the HCV DAAs and other chronic medication, using up to date resources such as the one found at http://www.hep-druginteractions.org [8].

The association in a single tablet of sofosbuvir (SOF), a nucleotide NS5B polymerase inhibitor, and ledipasvir (LDV), an inhibitor of nonstructural protein 5A (NS5A), is one of the possible treatment options for HCV genotypes 1 and 4, and has shown impressive results in co-infected patients both in clinical trials [11] and in real life cohorts [12]. This combination has few interactions with ARV and can be used with nonnucleoside reverse transcriptase inhibitors (NNRTIs), integrase inhibitors or ritonavir-boosted HIV protease inhibitors (PIs), excluding tipranavir [8, 10].

However, recent studies have raised concerns regarding the interaction between LDV and tenofovir (TDF). Although the mechanism is not completely understood, it is thought that LDV increases TDF levels through the inhibition of P-glycoprotein (P-gp) and breast cancer resistance protein (BCRP) [13]. Moreover, it has been shown that TDF exposure is even greater (30–60%) when SOF/LDV was co-administered with a boosted PI [14]. As administration of TDF with a boosted PI already increases TDF by 20–30%, most authors and guidelines recommend changing ARV or DAA therapy; if, however, this triple combination must be used, renal function should be closely monitored [13, 15–17]. In those with a glomerular filtration rate < 60 mL/min, these drugs should be avoided [13, 15–17].

The aim of our study was to evaluate the effect of the combination SOF/LDV in the eGFR in HCV/HIV patients who were receiving TDF as part of their antiretroviral therapy, and to analyze the added risk of the concomitant use of a boosted PI.

## Methods

This was a single center observational prospective study regarding all HCV/HIV patients treated with DAAs in our department. Data on baseline characteristics, co-morbidities, medication and evolution during HCV treatment was collected in a database that was previously approved by the hospital’s ethical commission, as well as by the National Data Protection Commission. Informed consent was obtained for all patients.

We analyzed the data of those patients who were treated with SOF/LDV for HCV and who had completed at least 12 weeks of follow up after treatment, from February 2015 to July 2017; patients who did not complete 12 weeks of follow up after treatment were excluded (death or abandonment) as well as patients with incomplete records. Patients were categorized in three groups according to their ARV regimen: (1) non TDF containing regimen (non TDF), (2) TDF without a boosted PI (non-boosted TDF) or (3) TDF with a ritonavir boosted PI (boosted TDF). Evolution of estimated glomerular filtration rate (eGFR) during and after HCV treatment was compared amongst the three groups. eGFR was calculated using the Chronic Kidney Disease Epidemiology Collaboration (CKD-EPI) formula.

Statistical analysis was performed using Statistical Package for the Social Sciences (SPSS®) software, version 23. Paired t- test was used for comparing baseline and end of treatment (EOT) mean eGFR evolution in each group, likelihood ratio was used for categorical variable analysis, ANOVA for analysis of mean difference within the 3 groups and Wilcoxon signed-rank test to evaluate the impact of cirrhosis in the eGFR. *P*-value < 0.05 was defined as statistically significant.

All patients in our cohort were evaluated for liver fibrosis using transient hepatic elastography (Fibroscan®) and, in some cases, liver biopsy. Patients with an hepatic elastrography value ≥12.5 KPa were considered cirrhotic.

## Results

From February 2015 until July 2017, 333 HCV/HIV coinfected patients were treated for chronic HCV infection in our department. Of these, 273 (81.9%) were prescribed the combination SOF/LDV for 12–24 weeks and completed 12 weeks of follow up. We excluded 19 patients from our analysis (three died during HCV treatment, three were lost to follow up and 13 had incomplete records).

All the 273 HCV/HIV patients were receiving antiretroviral medication: 53.1% (*n* = 145) received a non-TDF regimen, 28.6% (*n* = 78) used an unboosted TDF scheme and TDF plus boosted PI was administered in 18.3% (*n* = 50). Efavirenz was the most used third agent in both the non-TDF and unboosted-TDF groups (*n* = 50, 34,5% and *n* = 52, 66,7%, respectively); other drugs less used included rilpivirine (*n* = 2, 1,4% and *n* = 6, 7,7% respectively) and dolutegravir (*n* = 10, 6,9% and *n* = 1, 1,3%, respectively). Regarding the TDF + PI group, the most frequently used boosted PI was darunavir (*n* = 27, 54%), followed by atazanavir (*n* = 14, 28%) and finally lopinavir (*n* = 9, 18%); ritonavir was the booster used in all cases.

The baseline characteristics of the three groups (non-TDF, non-boosted TDF and with TDF + PI) are shown in Table 1. Almost all patients had undetectable HIV RNA (*n* = 258, 94.5%) and the mean CD4 cell count was 650/mm^3^ (±338/mm^3^).

**Table 1:** Baseline characteristics of patients according to ARV regimen

Baseline characteristics	Non TDF *n* = 145	Non-boosted TDF *n* = 78	TDF + Boosted PI *n* = 50	*p*
Male, *n*, (%)	127 (87.6)	67 (85.9)	45 (90.0)	0.78
Mean age ± SD (years)	47 ± 6	47 ± 6	45 ± 6	0.79
Baseline mean creatinine ± SD (mg/dL)	0.84 ± 0.19	0.8 ± 0.13	0.9 ± 0.14	0.05
Baseline mean eGFR ± SD (ml/min/1.73m^2^)	100.9 ± 16.3	104.1 ± 11.3	98.2 ± 11.8	0.06
Body mass index ± SD (Kg/m^2^)	23.3 ± 3.9	22.3 ± 4.3	22.8 ± 3.8	0.24
Diabetes,* n* (%)	5 (3.4)	4 (5.1)	1 (2.0)	0.63
Hypertension, *n* (%)	21 (14.5)	10 (12.8)	4 (8.0)	0.47
Median CD4^+^ cell count ± IQ (/mm^3^)	618 (457–868)	611 (443–762)	506 (279–807)	0.12
VL HIV < 20 cp/mL, *n* (%)	138 (95.2)	76 (97.4)	44 (88)	0.09
Median stiffness ± IQ (kPa)	9.4 (6.4–15.4)	9.5 (6.9–12.3)	10.5 (7.4–18.2)	0.74
Cirrhosis, *n* (%)	53 (36.6)	19 (24.4)	22 (44.0)	0.051
Median MELD ± IQ	7 (6–8)	7 (6–7)	7 (7–9)	0.07
Child-Pugh A,* n* (%)	33 (62.2)	8 (42.1)	9 (40.9)	0.10
SOF/LDV 24 weeks, *n *(%)	42 (27.2)	24 (30.0)	19 (38.0)	0.50
Ribavirin use,* n* (%)	28 (18.4)	14 (17.5)	9 (18.0)	0.96

*TDF* tenofovir, *PI* protease inhibitor, *Non TDF* regimens without TDF, *Non-boosted TDF* regimens with TDF without boosted PI, *TDF* Boosted PI – regimens with TDF and boosted PI, *SD* standard deviation, *IQ* interquartile range, *VL* viral load, *MELD* Model for End-Stage Liver Disease score, *SOF* sofosbuvir, *LDV* ledipasvir

**Fig. 1:**
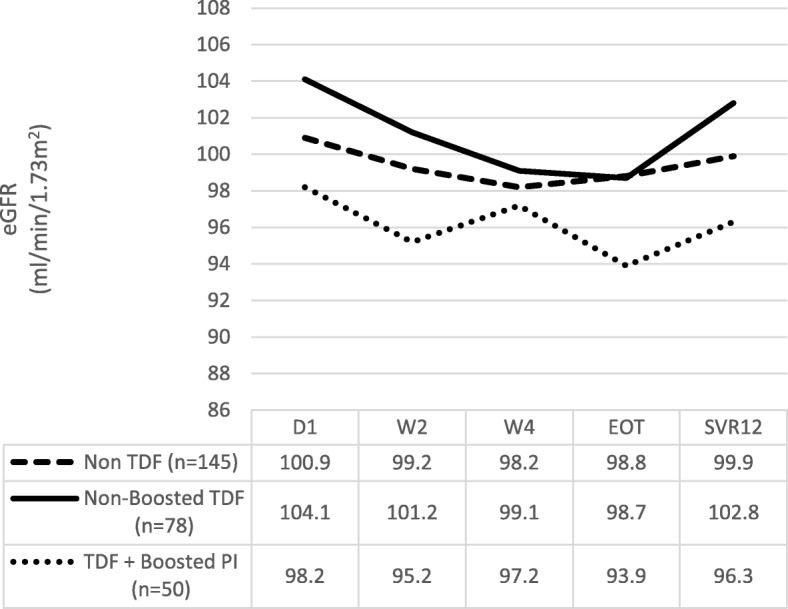
Evolution of the eGFR during treatment and at SVR12 according to ARV regimen. (eGFR – estimated glomerular filtration rate; D1 – day 1, beginning of treatment; W2 – week 2 of treatment; W4 – week 4 of treatment; EOT – end of treatment; SVR12 – end of follow up; TDF – tenofovir; PI – protease inhibitor; Non TDF – regimens without TDF; Non-boosted TDF – regimens with TDF without boosted PI; TDF + Boosted PI – regimens with TDF and boosted PI)

The mean baseline eGFR was 100.9 ml/min/1.73m^2^ (±16.3) in the non TDF group, 104.1 ml/min/1.73m^2^ (±11.3) in non-boosted TDF and 98.2 ml/min/1.73m^2^ (± 11.8) in the TDF + boosted PI group. In all groups, we observed a decrease in eGFR during treatment, as is shown in Fig. 1.

The decrease of eGFR between the baseline and the end of treatment (EOT) was statistically significant in all groups (non TDF *p* = 0.03, 95%CI [0.23–3.86], non-boosted TDF *p* < 0.01, 95%CI [3.36–7.44], TDF + PI *p* = 0.01, 95%CI [1.09–7.53]). The decrease was more pronounced in those receiving unboosted TDF (mean difference 5.40 ml/min/1.73m^2^; 95%CI [(− 7.44) -(− 3.37)]), than in those receiving either TDF + boosted PI (mean difference 4.31 ml/min/1.73m^2^; 95%CI [(− 7.53) -(− 1.09)] or non TDF regimens (mean difference 2.02 ml/min/1.73m^2^; 95%CI [(− 3.84) -(− 0.02)]). Differences in eGFR decrease between the three groups were small and were not statistically different (*p* = 0.06). (Table 2).

**Table 2:** Global Mean eGFR decrease between the baseline and end-of-treatment

Global Mean eGFR Decrease between the baseline and EOT		p	Confidence Interval
Non TDF *n* = 152	5.40 ml/min/1.73m^2^	0.03	95%CI [(−7.44) -(−3.37)]
Non-boosted TDF *n* = 80	4.31 ml/min/1.73m^2^	< 0.01	95%CI [(−7.53) -(− 1.09)]
TDF + Boosted PI *n* = 50	2.02 ml/min/1.73m^2^	0.01	95%CI [(− 3.84) -(− 0.02)]

*TDF* tenofovir, *PI* protease inhibitor, *Non TDF* regimens without TDF, *Non-boosted TDF* regimens with TDF without boosted PI, *TDF* Boosted PI – regimens with TDF and boosted PI, *EOT* end of treatment

At the end of follow up a recovery of eGFR was observed in all groups (Fig. 1).

The evolution of eGFR was similar between those who were treated for 12 weeks (*n* = 188) to those treated for 24 weeks (*n* = 85). However, there was a steeper decrease in eGFR in those who receiving a longer treatment course: mean change was − 2.1 95%CI [(− 3.43) -(− 7.96)] in those treated for 12 weeks and − 6.3 ml/min/1.73m^2^ 95%CI [(− 9.08) -(− 3.47)] in those treated for 24 weeks (*p* = 0.009). There was a statistical significant difference in mean eGFR decrease amongst the three ARV groups in those treated for 24 weeks (*p* = 0.026), but not in those who were treated for 12 weeks (*p* = 0.89). Of note, there was a higher proportion of cirrhotic patients in those treated for 24 weeks (81.2% of patients treated for this duration, *n* = 69) than in those treated for 12 weeks (13.3% of patients treated for 12 weeks, *n* = 25).

A separate analysis of the 94 cirrhotic patients included showed a similar evolution. There were no differences amongst the three groups (*p* = 0.833). However, cirrhosis was associated with a decrease in eGFR (*p* = 0.036): mean eGFR decrease for cirrhotic patients was − 5.57 ml/min/1.73m^2^ (95%CI [(− 8.16) -(− 2.98)]) vs − 2.28 ml/min/1.73 m^2^ (95%CI [(− 3.64) -(− 0.91)] for non-cirrhotic patients.

There were six patients whose eGFR decreased to less than 60 ml/min/1.73m^2^ during HCV treatment, but, except for one patient in the non-TDF group, these decreases were transient and were not sustained in the following assessments. The evolution of these patients is shown in Table 3.

**Table 3:** Evolution of the eGFR in patients with eGFR below 60 ml/min/1.73m^2^ during treatment

	Group	Gender, Age	Cirrhosis	HCV Treatment Duration	eGFR at baseline (ml/min/m^2^)	eGFR at W4 (ml/min/m^2^)	eGFR at EOT (ml/min/m^2^)	eGFR at SVR12 (ml/min/m^2^)
1	Non-TDF	Male, 66 yo	Yes	12w	59	45.3	56.9	63.9
2	Non-TDF	Male, 48 yo	Yes	24w	60	62.8	54.3	57.5
3	Non-TDF	Male, 49 yo	Yes	24w	65.9	57.1	37.8	32
4	Non-TDF	Male, 55 yo	Yes	12w	67.7	62	59.8	57.7
5	Non-boosted TDF	Male, 55 yo	Yes	24w	72.8	57.7	60.9	71.2
6	TDF+ Boosted PI	Male, 45 yo	Yes	24w	80.6	64.1	57.7	83.4

*eGFR* estimated glomerular filtration rate, *W2* week 2 of treatment, *W4*, week 4 of treatment, *EOT* end of treatment, *SVR12* end of follow up, *TDF* tenofovir, *PI* protease inhibitor, *Non-TDF* regimens without TDF, *Non-boosted TDF* regimens with TDF without boosted PI, *TDF* Boosted PI – regimens with TDF and boosted PI, *yo* years old, *w* week

No patient needed to interrupt HCV treatment due to renal dysfunction and no patient changed ARV during HCV treatment. The SVR12 was 98.6, 98.7 and 100% for the non-TDF, the unboosted TDF and boosted TDF group, respectively.

## Discussion

The SOF/LDV combination pill is an option for the treatment of HCV genotypes 1 and 4, due to its simple posology, good tolerability and few DDIs. However, international guidelines recommend caution (and frequent renal monitoring) when using these drugs in HIV coinfected patients receiving TDF and a booster (either ritonavir or cobicistat) as part of their ARV regimen due to the increased risk of TDF exposure and renal toxicity [15, 16].

In our cohort of SOF/LDV treated HCV/HIV co-infected patients, receiving multiple of ARV therapeutic schemes which included regimens without TDF, with TDF and TDF combined with a ritonavir boosted PI, we observed a significant decrease in eGFR during treatment in all study groups. The eGFR decrease was more pronounced in those patients receiving TDF in comparison with patients receiving either TDF with a boosted PI or non-TDF containing regimens, but the changes were small in all groups and had little clinical impact. Moreover, we found no statistical differences amongst the three groups regarding comorbidities. We found no explanation for the grater decrease in eGFR in the unboosted TDF group. Treatment duration had an impact in eGFR decrease, with longer treatment courses being associated with greater eGFR decrease. However, the proportion of cirrhotic patients was much higher in the group of patients treated for 24 weeks (81.2%) and cirrhosis was also associated with a decrease in eGFR (*p* = 0.036).

Despite this overall decrease, after treatment completion eGFR returned to almost baseline values in all groups. We observed the same occurrence in those who had a longer course of treatment and in cirrhotic patients. No patient needed to interrupt HCV treatment due to renal dysfunction and there were no ARV alterations; the SVR12 was above 98% in all groups.

Our results are consistent with the already published literature. Bhattacharya and colleagues analysed a cohort of 996 HCV genotype 1 and HIV co-infected patients, of which 895 were treated with SOF/LDV ± RBV; these authors did not find any difference in creatinine change during SOF/LDV treatment in those receiving either TDF containing ARV schemes (with or without PI) or those not receiving TDF [18]. Moreover, in line with our results, the median creatinine change was small (0.15 mg/dL in those without TDF, 0.18 mg/dL with TDF and 0.17 mg/dL in those with TDF + PI) and not different between the three groups (*p* = 0.30) [18].

Taramasso et al. evaluated the renal tolerability of the SOF/LDV in patients participating in the SCOLTA project (Surveillance Cohort Long-Term Toxicity Antiretrovirals), which included 79 HCV/HIV co-infected patients: 47 taking TDF, 34 a ritonavir boosted PI and 17 TDF + boosted PI [19]. The authors found no statistically significant variation of eGFR in patients receiving a boosted PI either in combination with TDF or not [19]. Moreover, they observed that patients receiving unboosted TDF experienced the highest percentage of filtration loss (− 5.3 mL/min (SD 15.8)) and that the frequency of eGFR loss > 5% was more frequent in this group but similarly to our results, there was no difference between those who took a boosted PI and those who did not [19].

Despite these reassuring results, Bunnell and colleagues published a case of acute tubular necrosis and acute interstitial nephritis in a HCV/HIV co-infected patient taking efavirenz/tenofovir/emtricitabine and receiving HCV treatment with SOF/LDV, that resolved after discontinuation of TDF and SOF/LDV [20]. Of note, the patient was also taking other medications that could contribute to renal injury [20].

The main limitations of our study are its observational nature and the lack of analysis of other parameters of renal lesion, namely urine analysis, proteinuria, albuminuria, as well as serum and urinary measurements of calcium and phosphate. Moreover, we did not evaluate the impact of other possible causes of nephrotoxicity. Additionally, data regarding HIV and HCV history is lacking, due to the lack of informatic records in our hospital.

The ongoing clinical trial NCT02588287 (Effects of Sofosbuvir/Ledipasvir Treatment on the Pharmacokinetics and Renal Safety of Tenofovir), promoted by the University of Colorado, is likely to shed some more light on the safety of this combination [21].

## Conclusion

We observed a decrease in the mean GFR in all patients treated with SOF/LDV. This decrease was higher in those receiving TDF, but the differences amongst the three groups were small and not statistically significant. Moreover, the eGFR decrease seems to be reversible after termination of HCV treatment. Our data, along with that of other authors [18, 19], reassures clinicians on the safety of SOF/LDV in combination with TDF containing regimens, regardless of the concomitant use of a boosted PI. However, from the authors point of view and as recommended in guidelines, if TDF is used in patients who are receiving SOF/LDV, signs of nephrotoxicity should be carefully monitored [15, 16], especially in cirrhotic patients.

## Abbreviations

ARV: Antiretroviral medication
DAA: Direct acting antiviral
DDIs: Drug-drug interactions
eGFR: estimated Glomerular filtration rate
EOT: End of treatment
HCV: Hepatitis C virus
HIV: Human immunodeficiency virus
LDV: Ledipasvir
NNRTI: Nonnucleoside reverse transcriptase inhibitor
PI: Protease inhibitor
SOF: Sofosbuvir
SVR: Sustained virologic response
TDF: Tenofovir
WHO: World Health Organization
